# The genome sequence of *Pycnococcus provasolii *(CCAP190/2) (Guillard, 1991)

**DOI:** 10.12688/wellcomeopenres.20345.1

**Published:** 2023-11-13

**Authors:** David H. Green, Cecilia Rad-Menéndez, Christine Campbell, Estelle S. Kilias, Culture Collection of Algae and Protozoa collective, University of Oxford and Wytham Woods Genome Acquisition Lab, Earlham Institute Genome Acquisition Lab and Protists Project, Darwin Tree of Life Barcoding collective, Wellcome Sanger Institute Tree of Life programme, Wellcome Sanger Institute Scientific Operations: DNA Pipelines collective, Tree of Life Core Informatics collective, Darwin Tree of Life Consortium

**Affiliations:** 1 Culture Collection of Algae and Protozoa, The Scottish Association for Marine Science, Oban, Scotland, UK; 2 Department of Biology, University of Oxford, Oxford, England, UK

**Keywords:** Pycnococcus provasolii, marine green alga, genome sequence, chromosomal, Pseudoscourfieldiales

## Abstract

We present a genome assembly from cultured *Pycnococcus provasolii* (a marine green alga; Chlorophyta; None; Pseudoscourfieldiales; Pycnococcaceae). The genome sequence is 32.2 megabases in span. Most of the assembly is scaffolded into 44 chromosomal pseudomolecules (99.67%). The mitochondrial and plastid genomes have also been assembled, and the length of the mitochondrial scaffold is 24.3 kilobases and of the plastid genome has been assembled and is 80.2 kilobases in length.

## Species taxonomy

Eukaryota; Viridiplantae; Chlorophyta; prasinophyte incertae sedis; Pycnococcaceae; *Pycnococcus*; *Pycnococcus provasolii* (Guillard, 1991) (NCBI:txid41880).

## Background

*Pycnococcus provasolii* is a marine green alga first described by Guillard *et al.* (1991) and that was one of the first planktonic eukaryotic species to be isolated from below the pycnocline ( Vaulot *et al.*, 2008). Despite the early isolation of *Pycnococcus* information on the genus is scarce to date.

*P. provasolii* is part of a group of small-sized coccoid algae that are gathered under the term prasinophytes, located at the base of the Chlorophyta, which is a sister group of the Streptophyta in Viridiplantae ( Lemieux *et al.*, 2014). Given the basal position of prasinophytes within the Chlorophyta, genomic data from members are important as they can hold key to understand the nature of the last common ancestor of all green plants.

Phylogenetic studies based on the SSU of 18S rDNA identified nine distinct lineages within the prasinophytes (clades I–IX) ( Guillou *et al.*, 2004; Viprey *et al.*, 2008). The paraphyletic origin of the group is reflected in a vast diversity of cell shapes and photosynthetic pigments ( Latasa *et al.*, 2004; Leliaert *et al.*, 2012; Lopes dos Santos *et al.*, 2017; Tragin *et al.*, 2016). Initially, *P. provasolii* was placed within the order of Mamiellales due to the presence of the photopigment prasinoxanthin, a diagnostic feature of the order at that time. The discovery of prasinoxanthin in another species, *Pseudoscourfielda marina* led to the formation of a new order Pseudoscourfieldiales with the family Pycnococcaceae, or prasinophyte clade V ( Lemieux *et al.*, 2014; Sym & Pienaar, 1993). Both species share a 18S rRNA gene identity of 100 percent ( Fawley *et al.*, 1999; Guillou *et al.*, 2004).

**Figure 1.**
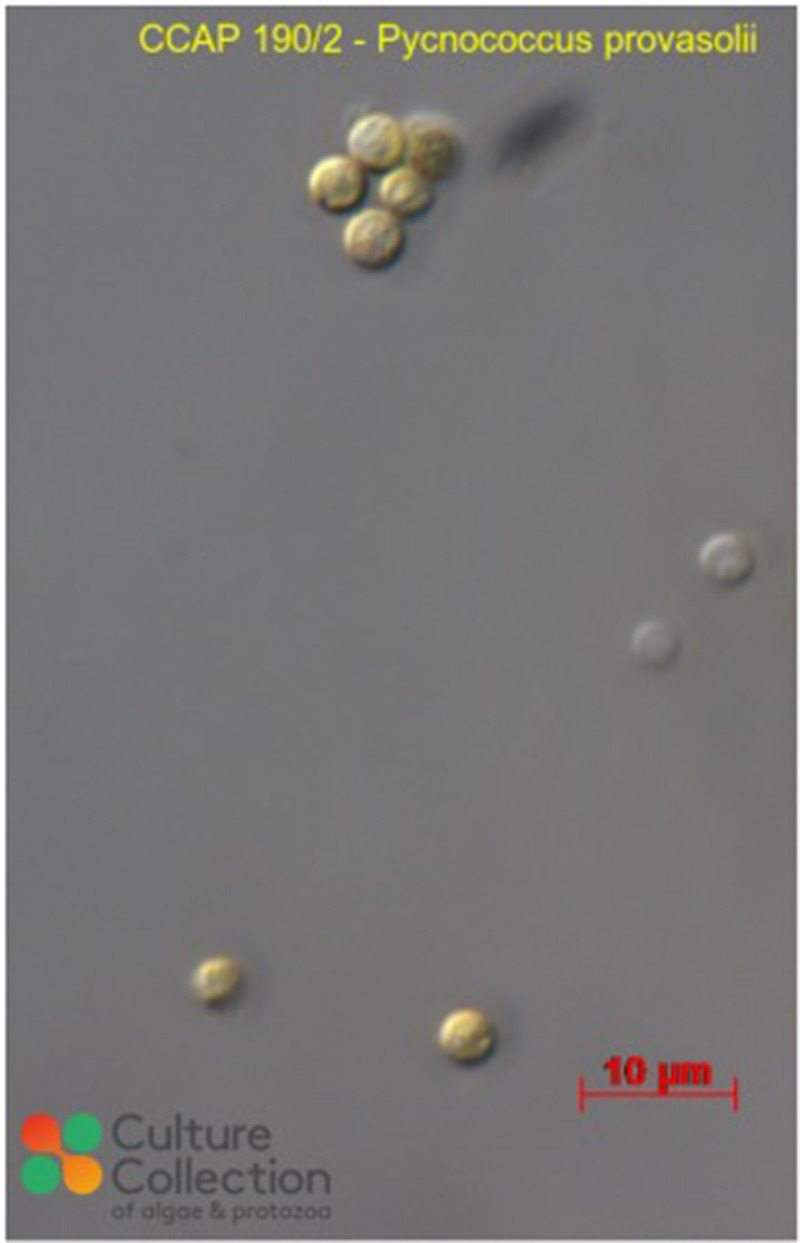
Light microscopy image of the strain 190/2 of *Pycnococcus provasolii*; available in the Culture Collection of Alae and protozoa (CCAP).

The morphology of *Pycnococcus provasolii* is described as a solitary coccoid cell of spherical shape with a size ranging between 1.5 and 4.0 μm in diameter ( Figure 1). The cell wall of *Pycnococcus provasolii* has the ultrastructural characteristics of green algae and lacks sporopollenin. Environmental DNA studies show a wide distribution pattern of *P. provasolii* covering different marine regions and light conditions, ranging between 0 and 100 m ( Viprey *et al.*, 2008; Zingone *et al.*, 2011). As a phototroph, sensitivity to light is crucial for survival. The photopigment composition of *P. provasolii* includes chl *a*, chl *b*, Mg 2,4-divinylphaeoporphyrin a _5_ monomethyl ester, and prasinoxanthin as a major xanthophyll ( Guillard *et al.*, 1991; Iriarte & Purdie, 1993). Further, a study by Makita *et al.* (2021) reported the presence of a bifunctional photoreceptor (PpDUC1) in the chloroplast genome, composed of phytochrome (PHY) and a cryptochrome (CRY). They hypothesise that the presence might have widen *P. provasolii*’s spectral utilisation.

The culture strain CCAP190/2 was isolated by C. Campbell in 2011 from Loch Scridian in Mull (Argyll, Scotland). Here we present the chromosomally complete genome of *P. provasolii* (190/2) which will help address a grand challenge in protists research, namely the lack of relevant genome sequences and help to understand the diversity and evolutionary history of the light response system in the Viridiplantae.

## Genome sequence report

The genome was sequenced from the strain CCAP190/2 of *Pycnococcus provasolii* ( Figure 1) maintained in culture by the Culture Collection of Algae and Protozoa, Oban, Scotland. A total of 444-fold coverage in Pacific Biosciences single-molecule HiFi long reads was generated. Primary assembly contigs were scaffolded with chromosome conformation Hi-C data. Manual assembly curation corrected 8 missing joins or mis-joins and removed 6 haplotypic duplications, reducing the scaffold number by 1.

**Figure 2.**
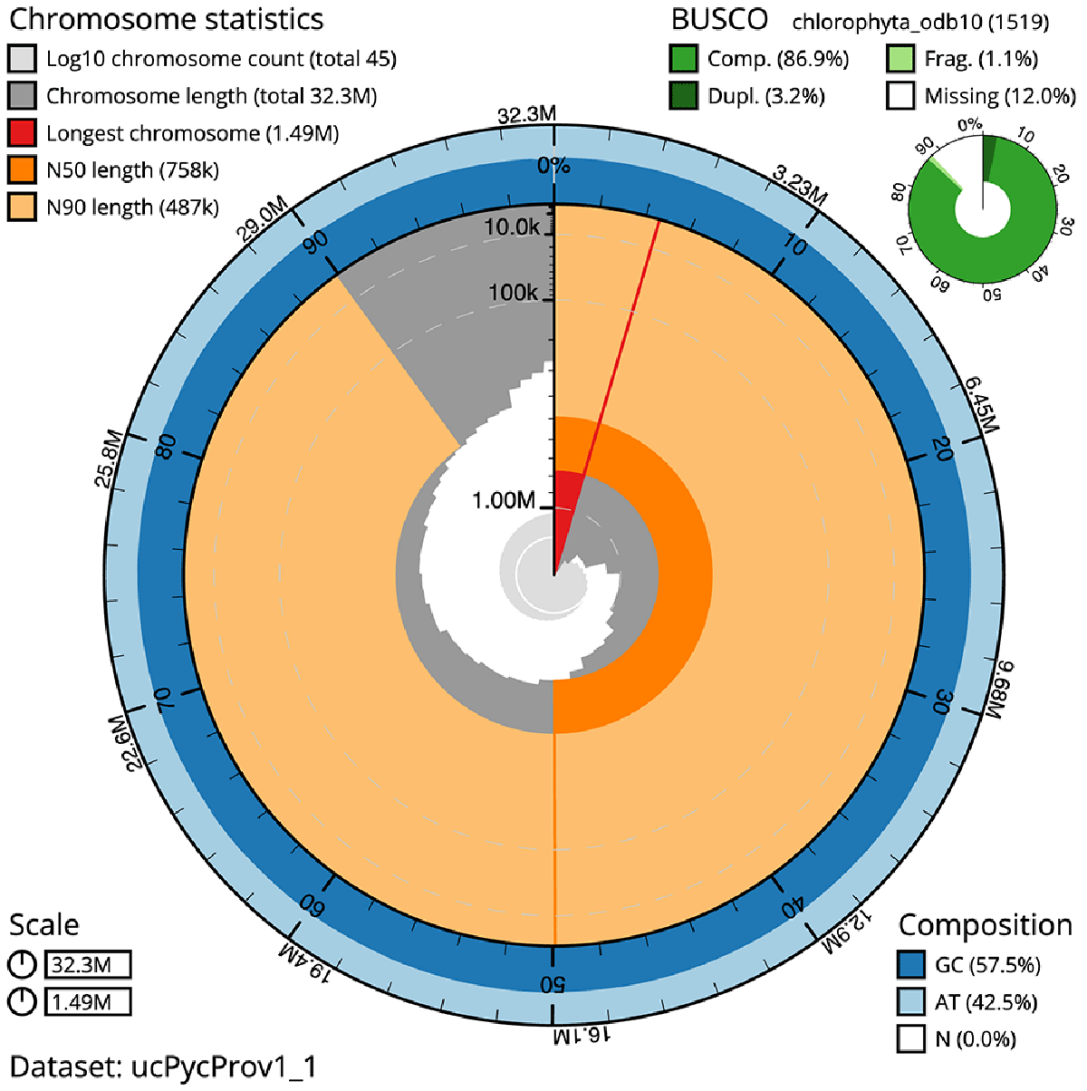
Genome assembly of *Pycnococcus provasolii*, ucPycProv1.2: metrics. The BlobToolKit Snailplot shows N50 metrics and BUSCO gene completeness. The main plot is divided into 1,000 size-ordered bins around the circumference with each bin representing 0.1% of the 32,253,916 bp assembly. The distribution of scaffold lengths is shown in dark grey with the plot radius scaled to the longest scaffold present in the assembly (1,490,684 bp, shown in red). Orange and pale-orange arcs show the N50 and N90 scaffold lengths (757,545 and 486,817 bp), respectively. The pale grey spiral shows the cumulative scaffold count on a log scale with white scale lines showing successive orders of magnitude. The blue and pale-blue area around the outside of the plot shows the distribution of GC, AT and N percentages in the same bins as the inner plot. A summary of complete, fragmented, duplicated and missing BUSCO genes in the chlorophyta_odb10 set is shown in the top right. An interactive version of this figure is available at https://blobtoolkit.genomehubs.org/view/Pycnococcus%20provasolii/dataset/ucPycProv1_1/snail.

**Figure 3.**
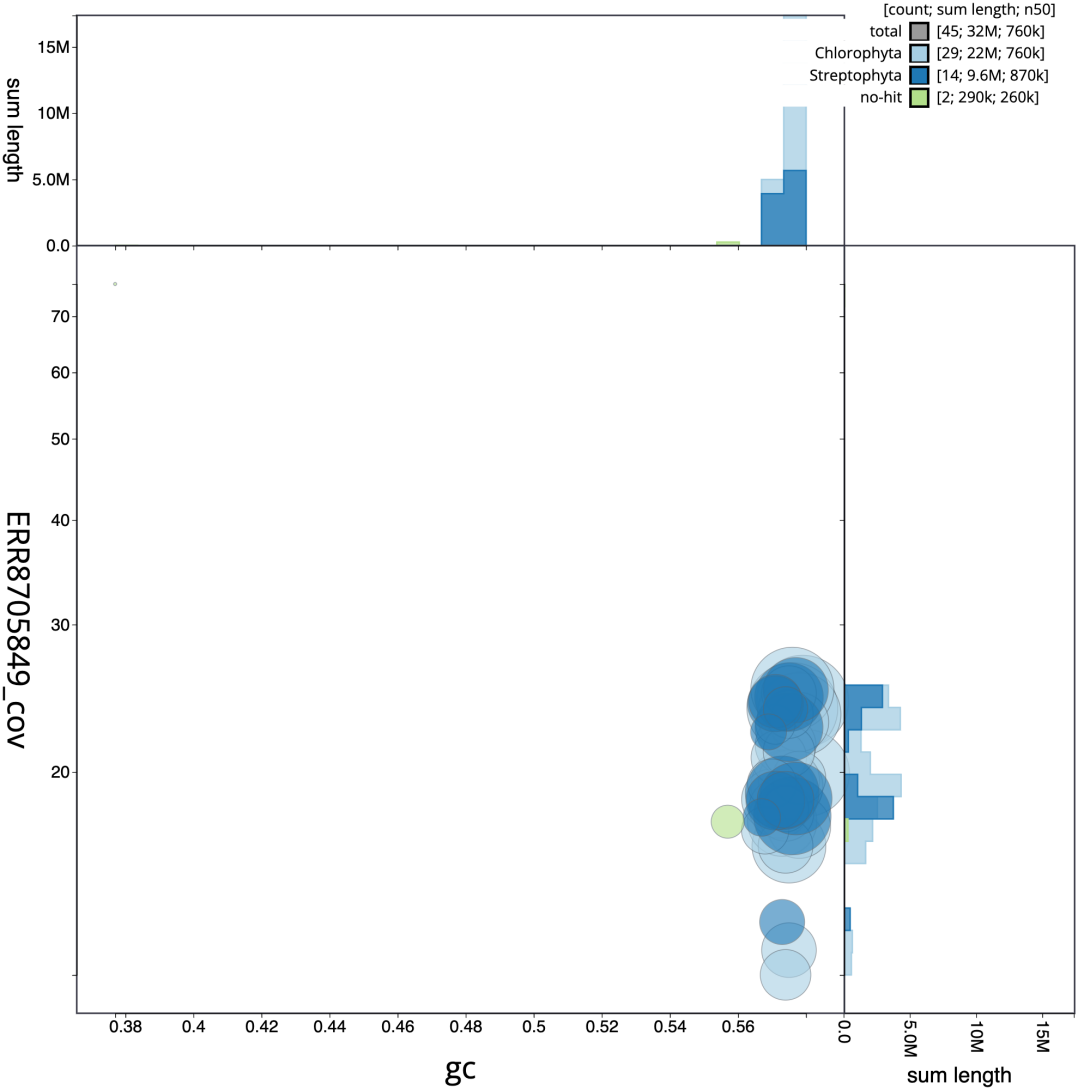
Genome assembly of *Pycnococcus provasolii*, ucPycProv1.2: BlobToolKit GC-coverage plot. Scaffolds are coloured by phylum. Circles are sized in proportion to scaffold length. Histograms show the distribution of scaffold length sum along each axis. An interactive version of this figure is available at https://blobtoolkit.genomehubs.org/view/Pycnococcus%20provasolii/dataset/ucPycProv1_1/blob.

**Figure 4.**
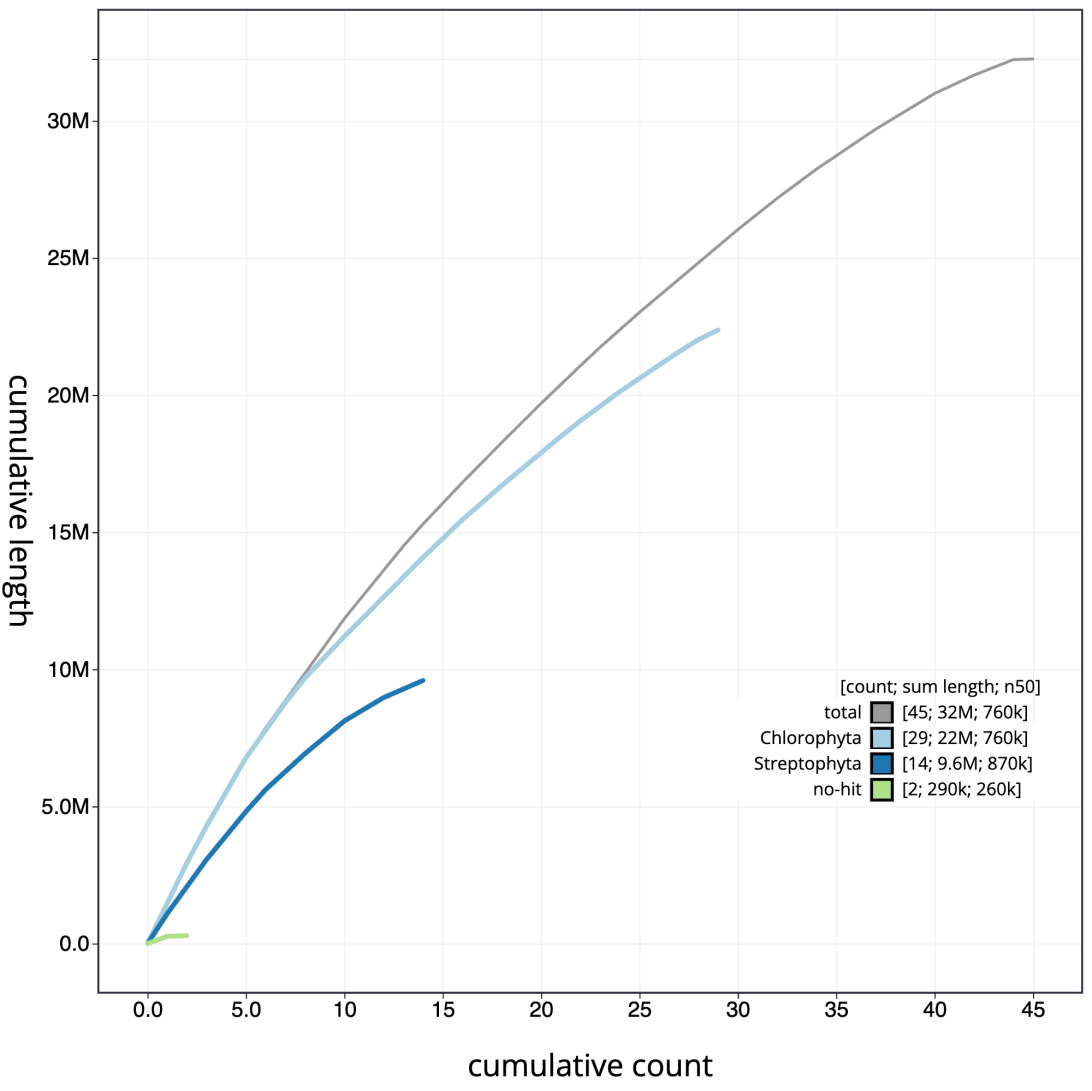
Genome assembly of *Pycnococcus provasolii*, ucPycProv1.2: BlobToolKit cumulative sequence plot. The grey line shows cumulative length for all scaffolds. Coloured lines show cumulative lengths of scaffolds assigned to each phylum using the buscogenes taxrule. An interactive version of this figure is available at https://blobtoolkit.genomehubs.org/view/Pycnococcus%20provasolii/dataset/ucPycProv1_1/cumulative.

**Figure 5.**
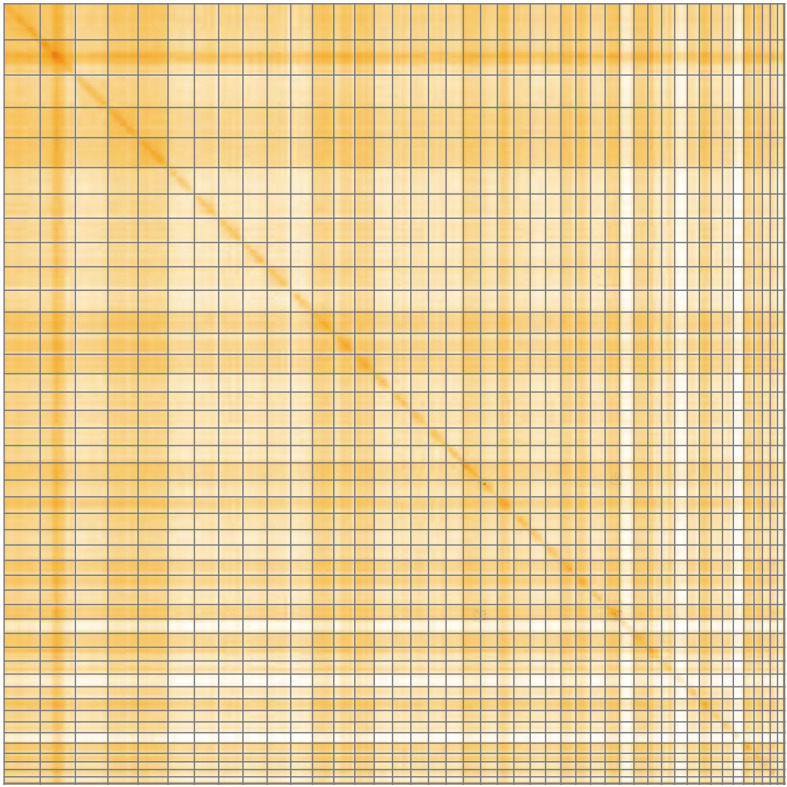
Genome assembly of *Pycnococcus provasolii*, ucPycProv1.2: Hi-C contact map of the ucPycProv1.2 assembly, visualised using HiGlass. Chromosomes are shown in order of size from left to right and top to bottom. An interactive version of this figure may be viewed at https://genome-note-higlass.tol.sanger.ac.uk/l/?d=PPdGu0uUSVObW9qATZ_0Ag.

The final assembly has a total length of 32.2 Mb in 44 sequence scaffolds with a scaffold N50 of 0.8 Mb ( Table 1). The snailplot in Figure 2 provides a summary of the assembly statistics, while the distribution of assembly scaffolds on GC proportion and coverage is shown in Figure 3. The cumulative assembly plot in Figure 4 shows curves for subsets of scaffolds assigned to different phyla. Most (99.67%) of the assembly sequence was assigned to 44 chromosomal-level scaffolds. Chromosome-scale scaffolds confirmed by the Hi-C data are named in order of size ( Figure 5; Table 2). Chromosomes 30 and 39 have roughly half coverage, which could be explained by the fact that some green algae can possess a mixture of diploid and haploid chromosomes. Telomeres have been identified on both ends of 36 of 44 chromosomes. While not fully phased, the assembly deposited is of one haplotype. Contigs corresponding to the second haplotype have also been deposited. The mitochondrial and plastid genomes were also assembled and can be found as a contig within the multifasta file of the genome submission.

**Table 1.** Genome data for *Pycnococcus provasolii*, ucPycProv1.2.

Project accession data		
Assembly identifier	ucPycProv1.2	
Species	*Pycnococcus provasolii*	
Specimen	ucPycProv1	
NCBI taxonomy ID	41880	
BioProject	PRJEB50458	
BioSample ID	SAMEA7524508	
Isolate information	ucPycProv1	
Assembly metrics *		*Benchmark*
Consensus quality (QV)	58.2	*≥ 50*
*k*-mer completeness	100%	*≥ 95%*
BUSCO **	C:86.9%[S:83.7%,D:3.2%], F:1.1%,M:12.0%,n:1,519	*C ≥ 95%*
Percentage of assembly mapped to chromosomes	99.67%	*≥ 95%*
Sex chromosomes	-	*localised homologous pairs*
Organelles	Mitochondrial and plastid genomes assembled	*complete single alleles*
Raw data accessions		
PacificBiosciences SEQUEL II	ERR8705849, ERR8705848	
Hi-C Illumina	ERR8571632	
Genome assembly		
Assembly accession	GCA_938743325.2	
*Accession of alternate haplotype*	GCA_938743315.1	
Span (Mb)	32.2	
Number of contigs	50	
Contig N50 length (Mb)	0.7	
Number of scaffolds	44	
Scaffold N50 length (Mb)	0.8	
Longest scaffold (Mb)	1.5	

* Assembly metric benchmarks are adapted from column VGP-2020 of “Table 1: Proposed standards and metrics for defining genome assembly quality” from ( Rhie *et al.*, 2021). ** BUSCO scores based on the chlorophyta_odb10 BUSCO set using v5.3.2. C = complete [S = single copy, D = duplicated], F = fragmented, M = missing, n = number of orthologues in comparison. A full set of BUSCO scores is available at https://blobtoolkit.genomehubs.org/view/Pycnococcus%20provasolii/dataset/ucPycProv1_1/busco.

**Table 2.** Chromosomal pseudomolecules in the genome assembly of *Pycnococcus provasolii*, ucPycProv1.

INSDC accession	Chromosome	Length (Mb)	GC%
OW568861.1	1	1.49	58.0
OW568862.1	2	1.45	57.5
OW568863.1	3	1.34	58.0
OW568864.1	4	1.25	58.0
OW568865.1	5	1.24	57.5
OW568866.1	6	1.09	57.5
OW568867.1	7	1.0	57.5
OW568868.1	8	1.0	57.5
OW568869.1	9	1.0	57.5
OW568870.1	10	0.97	57.5
OW568871.1	11	0.9	57.5
OW568872.1	12	0.88	57.5
OW568873.1	13	0.87	57.5
OW568874.1	14	0.8	57.5
OW568875.1	15	0.76	57.5
OW568876.1	16	0.76	58.0
OW568877.1	17	0.73	57.5
OW568878.1	18	0.73	57.5
OW568879.1	19	0.71	57.0
OW568880.1	20	0.71	57.5
OW568881.1	21	0.69	57.0
OW568882.1	22	0.68	58.0
OW568883.1	23	0.67	57.0
OW568884.1	24	0.64	57.5
OW568885.1	25	0.64	57.5
OW568886.1	26	0.62	57.0
OW568887.1	27	0.61	57.0
OW568890.1	30	0.6	57.5
OW568888.1	28	0.6	57.5
OW568889.1	29	0.6	57.5
OW568891.1	31	0.57	57.0
OW568892.1	32	0.57	58.0
OW568893.1	33	0.54	57.5
OW568894.1	34	0.52	57.5
OW568895.1	35	0.5	57.0
OW568896.1	36	0.49	57.5
OW568897.1	37	0.47	57.0
OW568898.1	38	0.45	57.5
OW568899.1	39	0.43	57.5
OW568900.1	40	0.42	57.5
OW568901.1	41	0.35	57.0
OW568902.1	42	0.32	56.5
OW568903.1	43	0.3	57.0
OW568904.1	44	0.26	55.5
OW568905.1	MT	0.02	38.0
OY726516.1	PLT	0.08	39.5

The estimated Quality Value (QV) of the final assembly is 58.2 with *k*-mer completeness of 100%, and the assembly has a BUSCO v5.3.2 completeness of 86.9% (single = 83.7%, duplicated = 3.2%), using the chlorophyta_odb10 reference set ( *n* = 1,519).

Metadata for specimens, spectra estimates, sequencing runs, contaminants and pre-curation assembly statistics can be found at https://links.tol.sanger.ac.uk/species/41880.

## Methods

### Sample acquisition and nucleic acid extraction

*Pycnococcus provasolii* (specimen ID SAN0000882, individual ucPycProv1) cells were isolated from a marine sample from Loch Scridian, Mull, Argyll, Scotland on 2011-01-01. The cells were collected and identified by Christine Campbell (CCAP). The *Pycnococcus provasolli* cells (ucPycProv1) were cultivated in f/2 media ( Guillard, 1975; Guillard & Ryther, 1962). The strain was incubated at 15˚C with a 12:12 h light:dark cycle at 5 µmol m ^–1^ s ^–1^. The cells were harvested by centrifugation and snap frozen in liquid nitrogen.

DNA was extracted at the Tree of Life laboratory, Wellcome Sanger Institute (WSI). The ucPycProv1 sample was weighed on dry ice with some of the sample set aside for Hi-C sequencing. The sample was cryogenically disrupted to a fine powder using a Covaris cryoPREP Automated Dry Pulveriser, receiving multiple impacts. High molecular weight (HMW) DNA was extracted using the Qiagen MagAttract HMW DNA extraction kit. HMW DNA was sheared into an average fragment size of 12–20 kb in a Megaruptor 3 system with speed setting 30. Sheared DNA was purified by solid-phase reversible immobilisation using AMPure PB beads with a 1.8X ratio of beads to sample to remove the shorter fragments and concentrate the DNA sample. The concentration of the sheared and purified DNA was assessed using a Nanodrop spectrophotometer and Qubit Fluorometer and Qubit dsDNA High Sensitivity Assay kit. Fragment size distribution was evaluated by running the sample on the FemtoPulse system. Protocols employed by the Tree of Life laboratory are publicly available on protocols.io: https://dx.doi.org/10.17504/protocols.io.8epv5xxy6g1b/v1.

### Sequencing

Pacific Biosciences HiFi circular consensus DNA sequencing libraries were constructed according to the manufacturers’ instructions. DNA sequencing was performed by the Scientific Operations core at the WSI on a Pacific Biosciences SEQUEL II (HiFi instrument. Hi-C data were also generated from the ucPycProv1 sample using the Arima2 kit and sequenced on the Illumina NovaSeq 6000 instrument.

### Genome assembly, curation and evaluation

The assembly process included the following sequence of steps: Tiara 1.0.1 ( Karlicki *et al.*, 2022) was run with PacBio HiFi reads. Reads classified as prokaryotic by Tiara were removed and the remaining reads were assembled using Hifiasm ( Cheng *et al.*, 2021) with default settings. The Hifiasm contigs were checked for contaminants using Tiara 1.0.1 and BLAST against NCBI nt and nr databases (May 2021 versions). Mitochondrial and plastid contigs were detected using Tiara. The longest organellar contigs were circularised using circlator minimus2 (1.5.5) ( Hunt *et al.*, 2015). The remaining copies of organellar contigs were discarded. Deduplication of chromosomal contigs was done using GAP5 (1.2.14-r) ( Bonfield & Whitwham, 2010). The assemblies were scaffolded with Hi-C Arima2 data using SALSA2 ( Ghurye *et al.*, 2019). The assembly was checked for contamination and corrected using the gEVAL system ( Chow *et al.*, 2016) as described previously ( Howe *et al.*, 2021). Manual curation was performed using gEVAL, HiGlass ( Kerpedjiev *et al.*, 2018) and Pretext ( Harry, 2022).

A Hi-C map for the final assembly was produced using bwa-mem2 ( Vasimuddin *et al.*, 2019) in the Cooler file format ( Abdennur & Mirny, 2020). To assess the assembly metrics, the *k*-mer completeness and QV consensus quality values were calculated in Merqury ( Rhie *et al.*, 2020). This work was done using Nextflow ( Di Tommaso *et al.*, 2017) DSL2 pipelines “sanger-tol/readmapping” ( Surana *et al.*, 2023a) and “sanger-tol/genomenote” ( Surana *et al.*, 2023b). The genome was analysed within the BlobToolKit environment ( Challis *et al.*, 2020) and BUSCO scores ( Manni *et al.*, 2021; Simão *et al.*, 2015) were calculated.

Table 3 contains a list of relevant software tool versions and sources.

**Table 3.** Software tools: versions and sources.

Software tool	Version	Source
BlobToolKit	4.0.7	https://github.com/blobtoolkit/blobtoolkit
BUSCO	5.3.2	https://gitlab.com/ezlab/busco
circlator minimus2	1.5.5	https://github.com/sanger-pathogens/circlator
gEVAL	N/A	https://geval.org.uk/
Hifiasm	0.12	https://github.com/chhylp123/hifiasm
HiGlass	1.11.6	https://github.com/higlass/higlass
Merqury	MerquryFK	https://github.com/thegenemyers/MERQURY.FK
MitoHiFi	2	https://github.com/marcelauliano/MitoHiFi
PretextView	0.2	https://github.com/wtsi-hpag/PretextView
purge_dups	1.2.3	https://github.com/dfguan/purge_dups
SALSA	2.2	https://github.com/salsa-rs/salsa
sanger-tol/genomenote	v1.0	https://github.com/sanger-tol/genomenote
sanger-tol/readmapping	1.1.0	https://github.com/sanger-tol/readmapping/tree/1.1.0
Tiara	1.0.1	https://github.com/ibe-uw/tiara

### Wellcome Sanger Institute – Legal and Governance

The materials that have contributed to this genome note have been supplied by a Darwin Tree of Life Partner. The submission of materials by a Darwin Tree of Life Partner is subject to the **‘Darwin Tree of Life Project Sampling Code of Practice’**, which can be found in full on the Darwin Tree of Life website here. By agreeing with and signing up to the Sampling Code of Practice, the Darwin Tree of Life Partner agrees they will meet the legal and ethical requirements and standards set out within this document in respect of all samples acquired for, and supplied to, the Darwin Tree of Life Project.

Further, the Wellcome Sanger Institute employs a process whereby due diligence is carried out proportionate to the nature of the materials themselves, and the circumstances under which they have been/are to be collected and provided for use. The purpose of this is to address and mitigate any potential legal and/or ethical implications of receipt and use of the materials as part of the research project, and to ensure that in doing so we align with best practice wherever possible. The overarching areas of consideration are:

•   Ethical review of provenance and sourcing of the material

•   Legality of collection, transfer and use (national and international)

Each transfer of samples is further undertaken according to a Research Collaboration Agreement or Material Transfer Agreement entered into by the Darwin Tree of Life Partner, Genome Research Limited (operating as the Wellcome Sanger Institute), and in some circumstances other Darwin Tree of Life collaborators.

## Data Availability

European Nucleotide Archive: *Pycnococcus provasolii*. Accession number PRJEB50458; https://identifiers.org/ena.embl/PRJEB50458 ( Wellcome Sanger Institute, 2022). The genome sequence is released openly for reuse. The *Pycnococcus provasolii* genome sequencing initiative is part of the Darwin Tree of Life (DToL) project. All raw sequence data and the assembly have been deposited in INSDC databases. The genome will be annotated using available RNA-Seq data and presented through the Ensembl pipeline at the European Bioinformatics Institute. Raw data and assembly accession identifiers are reported in Table 1.

## Author information

Members of the Earlham Institute Genome Acquisition Lab and Protists Project are given here: https://doi.org/10.5281/zenodo.8395242.

Members of the Culture Collection of Algae & Protozoa are given here: https://doi.org/10.5281/zenodo.7650573.

Members of the University of Oxford and Wytham Woods Genome Acquisition Lab are listed here: https://doi.org/10.5281/zenodo.4789928.

Members of the Darwin Tree of Life Barcoding collective are listed here: https://doi.org/10.5281/zenodo.4893703.

Members of the Wellcome Sanger Institute Tree of Life programme are listed here: https://doi.org/10.5281/zenodo.4783585.

Members of Wellcome Sanger Institute Scientific Operations: DNA Pipelines collective are listed here: https://doi.org/10.5281/zenodo.4790455.

Members of the Tree of Life Core Informatics collective are listed here: https://doi.org/10.5281/zenodo.5013541.

Members of the Darwin Tree of Life Consortium are listed here: https://doi.org/10.5281/zenodo.4783558.
