# Supplementary material for: The genome sequence of Pycnococcus provasolii (CCAP190/2) (Guillard, 1991)
Source: Wellcome Open Res. 2023 Nov 13;8:520. [Version 1] doi: 10.12688/wellcomeopenres.20345.1 (PMC11130579; doi:10.12688/wellcomeopenres.20345.1)
Supplement: Peer review history

# Reviewer report: Reviewer response for version 1

**DOI:** 10.21956/wellcomeopenres.22529.r81985

**Published:** 2024-05-27

**Authors:** Senjie Lin

**Affiliations:** 1 Department of Marine Sciences, University of Connecticut, Storrs, Connecticut, USA

This is the second strain of Pycnococcus provasolii, the genome of which has been sequenced, and a valuable addition to the growing algal genomic database. Previously, Makita et al. (2021; https://doi.org/10.1038/s41467-021-23741-5) sequenced the ~22.7 Mbp genome of *P. provasolii* strain NIES-2893 using paired-end and mate-pair short reads and nanopore long reads. The reads were assembled to 43 scaffolds. From the genome 11,297 genes were predicted, with 89.1% (270/303) BUSCO completeness. This second genome was sequenced using PacBio single-molecule HiFi long-read sequencing coupled with Hi-C analysis. The genome was assembled to 32.2 Mbp in 44 scaffolds with a length ranging from 0.26 to 1.49 Mbp and a N50 of 0.8 Mbp. The assembly exhibited 86.9% BUSCO completeness. The sequencing technology in the newer work is clearly more of cutting edge from today’s point of view, but the older work also did a great job and achieved a reasonable assembly, in fact a slightly higher BUSCO completeness than the newer study.

 The larger genome size and one more chromosome in strain CCAP190/2 than in strain NIES-2893 suggest remarkable genomic differentiation between intraspecific strains.

 Besides the nuclear genome, the newer work by Green and colleagues assembled the mitochondrial and chloroplast genomes. They were 24 and 80 Kbp long, respectively, positioned towards the small end of algal mitogenomes and plastid genomes, but consistent to prasinophyte’s small cell size and small nuclear genome. The organelle genomes are a nice addition.

 It would be nice to see a comparison of the mitogenomes and plastid genomes with that of the other strain and information about whether the organelle genomes are as much differentiated between strains as the nuclear genome. The lack of such comparison appears to be due to the unavailability of organelle genomes for stain NIES-2893. Nevertheless, some evolutionary insights between these organelle genomes and organelles of other algal lineages would make the data shine much more.

 Besides, I do not seem to be able to find gene numbers predicted from the CCAP190/2 nuclear genome. The number and its comparison with the other strains are highly desirable.

 In addition, it is curious that the nuclear genome assemblies from the two sequenced strains were not compared in the current report. To me, this constitutes a major shortfall of the report, even though it is a data report. In particular, a systematic synteny analysis would shed light on how much similarity and dissimilarity occur in terms of the organization of DNA between the strains.

 Finally, the previous genomic work has revealed a dual photoreceptor containing phytochrome and cryptochrome, initially from metagenome and subsequently confirmed by the genome data of strain NIES-2893 (Makita et al. 2021). Does this also occur in this CCAP190/2 strain? If so, are there differences in its organization, copy number, and localization in the chromosomes?

 Conceivably, a research paper might be underway providing more complete comparative genomics information and insights into evolution and ecological niche differentiation. I look forward to such a paper.

Are sufficient details of methods and materials provided to allow replication by others?

Yes

Is the rationale for creating the dataset(s) clearly described?

Yes

Are the datasets clearly presented in a useable and accessible format?

Yes

Are the protocols appropriate and is the work technically sound?

Yes

Reviewer Expertise:

Genomics, molecular biology, and ecology of algae

I confirm that I have read this submission and believe that I have an appropriate level of expertise to confirm that it is of an acceptable scientific standard.

## References

[1] : Identification of a dual orange/far-red and blue light photoreceptor from an oceanic green picoplankton. *Nat Commun* .2021;12(1) : 10.1038/s41467-021-23741-5 3593 10.1038/s41467-021-23741-5 34135337 PMC8209157

# Reviewer report: Reviewer response for version 1

**DOI:** 10.21956/wellcomeopenres.22529.r70026

**Published:** 2024-05-25

**Authors:** Jason E. Stajich

**Affiliations:** 1 Department of Microbiology and Plant Pathology, University of California Riverside, Riverside, USA

This genome report provides clear description for the importance of this marine algae, the approaches used to grow the culture, obtain DNA, genome sequencing and assembly, and assessment of the quality of the completed genome assembly. 

 The culture provenance is clearly described and the primary short read data are all available as part of the data deposit in EMBL databases.

 Perhaps only places for additional detail would be

- How long was the culture grown for in order to obtain the cell density needed for the extraction?
- The methods / pattern used to identify the telomere sequence are not described specifically or if this repeat sequence is a common one or determined based on this dataset
- Do the authors feel the lower completeness of the BUSCO (12% missing) is really a function of genes lost in the green algae in general and not present in the chlorophyta_odb10 marker set?

But these are all minor points and this is generally a well written and complete genome announcement report.

Are sufficient details of methods and materials provided to allow replication by others?

Yes

Is the rationale for creating the dataset(s) clearly described?

Yes

Are the datasets clearly presented in a useable and accessible format?

Yes

Are the protocols appropriate and is the work technically sound?

Yes

Reviewer Expertise:

Genomics, systematics, bioinformatics and genome assembly and annotation.

I confirm that I have read this submission and believe that I have an appropriate level of expertise to confirm that it is of an acceptable scientific standard.
